# Comparative Analysis of Human Umbilical Cord Blood-Derived Mesenchymal Stem Cells between Preeclampsia and Normal Pregnant Women

**DOI:** 10.1155/2020/8403192

**Published:** 2020-06-08

**Authors:** Han-Sung Hwang, Yong-Sun Maeng

**Affiliations:** ^1^Department of Obstetrics and Gynecology, Konkuk University Medical Center, Konkuk University School of Medicine, Seoul, Republic of Korea; ^2^Department of Obstetrics and Gynecology, Institute of Women's Life Medical Science, Yonsei University College of Medicine, Seoul, Republic of Korea

## Abstract

Preeclampsia is a syndrome characterized by deterioration of either the maternal condition or the fetal condition. The adverse intrauterine environment made by preeclampsia results into intrauterine growth restriction and increased risk of a variety of diseases in future life. Given the adverse environment of fetal circulation made in the preeclamptic condition, and the role of mesenchymal stem cell (MSC) as a multipotent progenitor cell, we hypothesized that MSCs derived from human umbilical cord blood (hUCB-MSCs) obtained from preeclampsia are adversely altered or affected compared with normal pregnancy. The aim of this study was to analyze the biological characteristics and compare the functional abilities and gene expression patterns of hUCB-MSCs originating from pregnant women with and without severe preeclampsia. hUCB-MSCs were isolated and cultured from 28 pregnant women with severe preeclampsia and 30 normal pregnant women. hUCB-MSCs obtained from women with preeclampsia were less proliferative and more senescent and had lower telomerase activity and higher ROS activity than cells from women with normal pregnancy. In addition, many senescence-related differentially expressed genes (DEGs) were identified by analysis of microarray gene expression profiles and significantly associated with the Gene Ontology term cell aging. In conclusion, hUCB-MSCs obtained from women with preeclampsia showed the poorly proliferative, more senescent, and decreased telomerase activity, and these characters may be related with functional impairment of MSC from preeclampsia compared with cells from normal pregnancy.

## 1. Introduction

The discovery of mesenchymal stem cells (MSCs) by Friedenstein et al. in 1976 suggested a potentially useful model for gene therapy, regenerative medicine, and better and more advanced treatment strategies for various diseases, even those that seem to be incurable [1]. An increasing number of reports indicate that MSCs have extensive proliferative potential and the ability to differentiate into various cell types, including osteoblastic, adipogenic, chondrogenic, myogenic, and neurogenic cells [2–5]. Because of these properties, numerous laboratories are studying the clinical safety and efficacy of MSCs for the treatment of a number of pathological conditions, such as heart failure [6], spinal cord injury [7], and bone and cartilage diseases [8]. Whereas bone marrow was the first main source of MSCs, recent studies have suggested that MSCs can be obtained from many other tissues of the human body, such as fat [9], umbilical cord blood, chorionic villi of the placenta [10], amniotic fluid [11], peripheral blood [12], lung [13], skeletal muscle [14], synovial membrane [15], hepatic tissue [16], and even exfoliated deciduous teeth [17]. In particular, recent studies showed that MSCs derived from human umbilical cord blood (hUCB-MSCs) could be isolated more efficiently and are more developmentally primitive than MSCs derived from adult tissues [18]. For hematopoietic stem cells derived from umbilical cord blood, the various senescent stages and their regulatory pathways are well known [19–21]. In contrast, the mechanisms of senescence and functional impairment of MSCs remain unknown, although several recent studies have shown that MSCs isolated from older donors are more senescent than those isolated from younger donors [22, 23] and that MSCs have a replicative senescence pathway involving intracellular superoxide accumulation [24, 25].

Preeclampsia is a complication found in 2-8% of pregnancies and a major cause of maternal and perinatal morbidity and mortality [26–30]. Preeclampsia is a syndrome characterized by deterioration of either the maternal condition (hypertension and proteinuria with or without multiorgan abnormalities) or the fetal condition (intrauterine growth restriction, decreased amniotic fluid) [31–33]. Intrauterine growth restriction is a major fetal complication of preeclampsia. Although reduced placental blood flow [34, 35] and increased sensitivity of the human placental vasculature to vasoconstrictors have been suggested as possible causes [36], the pathophysiology of intrauterine growth restriction in preeclampsia is still unclear. Moreover, children born at term to mothers with preeclampsia have an increased risk of a variety of diseases, such as endocrine, nutritional, and metabolic diseases, as well as diseases of the blood and blood-forming organs [37]. These findings in the preeclamptic condition may originate through adaptations of the fetus to an adverse intrauterine environment. Previous studies have given explanations for this adverse condition comparison of umbilical cord blood with and without preeclampsia. As compared with the normal pregnancy group, increased antiangiogenic factors, reduced expression of proangiogenic signal, elevated oxidative stress, and increased inflammatory response have been founded in fetal serum during preeclampsia [38–40].

Given the adverse environment of fetal circulation made in the preeclamptic condition, and the role of MSC as a multipotent progenitor cell, we hypothesized that hUCB-MSCs obtained from preeclampsia are adversely altered or affected compared with normal pregnancy. The aim of this study was to analyze the biological characteristics and compare the functional abilities and gene expression patterns of hUCB-MSCs originating from pregnant women with and without severe preeclampsia.

## 2. Materials and Methods

### 2.1. Study Participants and Sample Collection

We studied the cord blood of pregnant women who visited Konkuk University Hospital, 30 of whom had no pregnancy complications (normal group) and 28 of whom had severe preeclampsia (preeclampsia group). Only women delivered by a cesarean section without labor were enrolled in this study. Umbilical cord blood was obtained from each pregnant woman at the time of the cesarean section. The indications of the cesarean section for pregnant women without preeclampsia were a previous cesarean section, previous myomectomy, breech presentation, or transverse lie. All subjects were enrolled in this study after signing an informed consent document approved by the institutional review board (IRB No: KUH1040005). Severe preeclampsia was defined as the presence of hypertension (systolic blood pressure ≥ 160 mmHg and/or diastolic pressure ≥ 110 mmHg) and proteinuria (≥3+ on a dipstick test or 5 g per 24 hours) beyond the 20^th^ week of pregnancy [41]. At least two consecutive measurements were required for diagnosis. Subjects were excluded from this study if they had known fetal or maternal complications, such as multiple gestation, fetal structural or genetic problems, maternal chronic hypertension, cardiovascular disease, renal disease, hepatic disease, diabetes mellitus, infectious disease, connective tissue disease, and autoimmune disease.

### 2.2. Isolation and Cultivation of hUCB-MSCs

Umbilical cord blood samples (about 50 mL each) with EDTA as an anticoagulant were collected from an umbilical cord vein attached to the placenta by gravity flow after delivery. Mononuclear cells (MNCs) were isolated from cord blood samples by density gradient centrifugation over Biocoll (Biochrom, Berlin, Germany) for 30 minutes at 400 × *g* and washed three times in phosphate-buffered saline (PBS) (Biochrom). Among the MNCs, CD133/c-kit-positive cells were selected to differentiate into MSCs. CD133/c-kit-positive cells were enriched using the MACS system (Miltenyi Biotec, Bergisch Gladbach, Germany). Briefly, MNCs were washed and resuspended in PBS buffer. Cells were incubated with anti-CD133/c-kit microbeads in the presence of human IgG as a blocking reagent at 4°C for 30 min. Labeled cells were loaded onto a column installed in a magnetic field. The column was rinsed with PBS buffer, and negative cells passed through. Trapped cells were eluted after the removal of the column from the magnet. Isolated CD133/c-kit positive cells were seeded 1 × 10^6^ cells on 6-well plates, coated with human fibronectin (Sigma-Aldrich Chemie, Munich, Germany) in endothelial basal medium-2 (EBM-2) (Clonetics, Cell Systems, St. Katharinen, Germany). The medium was supplemented with endothelial growth medium-2 (EGM-2; Clonetics, Cell Systems) containing fetal bovine serum, human VEGF-A, human fibroblast growth factor-B, human epidermal growth factor, insulin-like growth factor 1 (IGF1), and ascorbic acid. After 3 days, nonadherent cells were removed and fresh culture medium was added. Cultures were maintained with EGM-2 supplement. Phenotypical analysis of the cells was performed on days 7, 13, and 15.

### 2.3. Flow Cytometric Analysis

The MSCs were treated with 0.25% trypsin, washed once with PBS, and collected. The following fluorescently labeled antibodies were used for flow cytometric characterization of hUCB-MSCs: anti-CD29 (Molecular Probes, Eugene, OR), anti-CD73 (BD Pharmingen, San Diego, CA), and anti-CD90 (Abcam, Cambridge, MA**)**. Detached cells were washed twice with Dulbecco's PBS, centrifuged, washed in ice-cold Dulbecco's PBS supplemented with 1% bovine serum albumin (FCM buffer), and fixed in 2% paraformaldehyde in FCM buffer. Cells were then incubated with antibodies conjugated to fluorescein isothiocyanate (FITC) or phycoerythrin (PE) (Pharmingen, BD Biosciences Europe, Heidelberg, Germany) for 15 minutes on ice in a dark room at concentrations recommended by the manufacturer. We used anti-IgG-FITC (33814X; Pharmingen, BD Biosciences Europe) and anti-IgG-PE (33815X; Pharmingen, BD Biosciences Europe) as isotypic controls. After being washed, cells were analyzed on a Coulter EPICS XL-MCL flow cytometer (Beckman Coulter, Krefeld, Germany) by using EXPO-32 software. Instrument settings for scatter conditions and background fluorescence were adjusted for untreated cells.

### 2.4. Semiquantitative Reverse Transcription Polymerase Chain Reaction (RT-qPCR)

Total RNA was extracted from cells with a TRIzol reagent kit (Invitrogen, Carlsbad, CA) according to the manufacturer's instructions. Briefly, first-strand complementary DNA (cDNA) was synthesized from 1 *μ*g of total RNA with SuperScript II reverse transcriptase, oligo (dT) primer, and 10 mM dNTP mixture (Invitrogen, Carlsbad, CA). The cDNA mixture (1 *μ*L) was used for PCR. Amplification of the GAPDH gene was carried out in parallel for normalization. The PCR was performed in a DNA thermal cycler (model PTC-200; MJ Research, Scientific Support, Inc., Waltham, MA) under the following conditions: denaturation at 94°C for 5 minutes for the first cycle and for 30 seconds thereafter, annealing at 60°C for 30 seconds, and extension at 72°C for 30 seconds for 40 cycles. All results were normalized to GAPDH mRNA. The primers used are given in Table 1.

### 2.5. Immunofluorescence Staining

MSCs were put on glass coverslips at the bottom of wells in 24-well culture plates, fixed with 4% formaldehyde solution, and permeabilized with 0.3% Triton X-100. They were incubated with 3% hydrogen peroxide in methanol for 10 minutes to block endogenous peroxidase activity and then washed twice in PBS for 5 minutes. Cells were incubated overnight at 4°C with the following primary antibodies: mouse anti-human monoclonal antibody against *α*-SMC (Sigma-Aldrich, St. Louis, MO), 1 : 200; mouse anti-human monoclonal antibody against CD90 (Abcam, Cambridge, MA), 1 : 100; and mouse anti-human monoclonal antibody against CD73 (BD Pharmingen, San Diego, CA), 1 : 500. Cells were washed three times with PBS containing Triton X-100 and mounted with Vectashield mounting medium containing 4′,6-diamidino-2-phenylindole (DAPI) (Vector Laboratories). After being washed twice for 15 minutes in PBS, slides were incubated (30 minutes at 37°C) with secondary antibody (anti-goat IgG antibody conjugated to Alexa 488 or 555; Molecular Probes) and then with streptavidin-conjugated horseradish peroxidase. Slides were developed with 3,3′-diaminobenzidine tetrahydrochloride.

### 2.6. Osteogenic Differentiation

To induce osteogenic differentiation, we plated MSCs in a 24-well plate at 5 × 10^3^ cells/cm^2^. At 70% confluency, cells were treated with osteogenic induction medium (low-glucose Dulbecco's modified Eagle's medium, 10% FBS, 10 mm *β*-glycerophosphate, 10 nm dexamethasone, 50 *μ*m ascorbate, and antibiotics). The medium was changed every 3-4 days, and cell morphology was assessed visually every day for up to 3 weeks. At the end of differentiation, cells were stained for alkaline phosphatase and with Von Kossa stain [42–44].

### 2.7. Adipogenic Differentiation

To induce adipogenic differentiation, we treated MSCs with adipogenic medium (1 *μ*M dexamethasone, 0.5 mM 3-isobutyl-1-methylxanthine, 10 *μ*g/mL recombinant human insulin, 0.2 mM indomethacin, and 10% fetal calf serum) for 3 weeks. Maintenance medium including only recombinant human insulin and 10% fetal calf serum was replaced twice weekly, and adipogenesis was assessed at weekly intervals. Control cells were kept in adipogenic maintenance medium. Cells were fixed with 10% formalin, washed, and stained with 0.18% Oil Red O solution for 5 minutes. Adipogenic differentiation was confirmed by intracellular accumulation of lipid-rich vacuoles that stained with Oil Red O [43–45].

### 2.8. Chondrogenic Differentiation

To induce chondrogenic differentiation, we cultured 3 × 10^5^ MSCs/well in chondrogenic medium (high-glucose Dulbecco's modified Eagle's medium, 1x insulin-transferrin-selenium premix, 0.1 mM ascorbic acid 2-phosphate, 10 mM sodium pyruvate, 10 ng/mL transforming growth factor-*β*1, and 100 nM dexamethasone) for three weeks. Medium changes were carried out twice weekly, and chondrogenesis was assessed at 2~3-day intervals. Cells were fixed in 4% formaldehyde, dehydrated in an ethanol series, and embedded in paraffin blocks. Blocks were cut, and sections were stained for sulphated proteoglycans with Safranin O (0.1% aqueous solution) (Sigma-Aldrich) to evaluate chondrogenic differentiation [43, 44].

### 2.9. Proliferation Assay

To compare the doubling time of hUCB-MSCs between groups, cells were seeded in six T-25 flasks. On each of six consecutive days, the cells from one flask were obtained and enumerated. Mean counts were calculated. The mean population doubling time (PD) was calculated with the following formula: PD = *t* × lg2/(lg*N*_*t*_ − lg*N*_0_), where *N*_0_ is the inoculum cell number, *N*_*t*_ is the number of harvested cells, and *t* is the duration of culture (in hours) [46].

### 2.10. Senescence-Associated *β*-Galactosidase Assay

The senescence-associated *β*-galactosidase (SA-*β*-gal) assay was performed to distinguish senescent cells [47]. SA-*β*-gal activity of hUCB-MSCs at passage 3 was measured and compared between two groups. Briefly, hUCB-MSCs were washed in PBS, fixed for 3 minutes (at room temperature) in 2% paraformaldehyde, washed, and incubated for 24 hours at 37°C with fresh SA-*β*-gal staining solution (1 mg/mL 5-bromo-4-chloro-3-indolyl *β*-D-galactopyranoside, 5 mM potassium ferrocyanide, 5 mM potassium ferricyanide, 150 mM NaCl, 2 mM MgCl_2_, 0.01% sodium deoxycholate, and 0.02% Nonidet P-40). hUCB-MSCs were counterstained with DAPI (0.2 *μ*g/mL in 10 mM Tris-HCl, pH 7.0, 10 mM EDTA, and 100 mM NaCl) for 10 minutes. Distinctly stained cells were observed by phase contrast microscopy. The mean staining intensity of SA-*β*-gal-positive cells was calculated from four randomly selected microscopic fields (×200 magnification) by densitometry [48].

### 2.11. Telomerase Activity Assay

To analyze the telomerase activity of hUCB-MSCs quantitatively, we conducted a telomeric repeat amplification protocol assay using a TeloTAGGG Telomerase PCR ELISA kit (Roche Molecular Biochemicals, Brussels, Belgium) according to the manufacturer's protocol. The telomerase activity of hUCB-MSCs at passage 3 was measured and compared between two groups. Briefly, 2 × 10^5^ hUCB-MSCs were pelleted at 3000 *g* for 10 minutes at 4°C, washed twice with cold PBS, incubated for 20 minutes at 4°C with 200 *μ*L of precooled lysis buffer (solution 1 of the kit), and centrifuged at 16,000 *g* for 20 minutes. Telomeric repeats were added to a biotin-labeled primer during the first reaction, and then, the elongation products were amplified by PCR. Finally, the immobilized PCR product was detected with an anti-digoxigenin-peroxidase antibody and visualized as a colored reaction product with the substrate 3,3′,5,5′-tetramethyl benzidine. The absorbance was measured in triplicate at 450 nm, by reading against a blank (reference absorbance at 690 nm). Samples were regarded as telomerase-positive if the difference in absorbance (*A*_450_ − *A*_690_) was greater than 0.2 [49, 50].

### 2.12. Reactive Oxygen Species (ROS) Activity Assay

Endogenous superoxide production was evaluated using the oxidative fluorescent dye dihydroethidium (DHE). ROS activity of hUCB-MSCs at passage 3 was measured and compared between two groups. Cells were plated on 12-well plates, washed with Krebs-HEPES buffer (pH 7.4), and stained with DHE for 15 minutes at 37°C in an incubator. After fixation with paraformaldehyde, slides were coverslipped with mounting medium and photos were taken [51, 52].

### 2.13. Densitometric Analysis

SA-*β*-gal-positive cells and DHE-stained cells were visualized by densitometric scanning using a luminescent image analyzer (LAS-1000, Fuji Photo Film Co. Ltd., Tokyo, Japan) and digital analysis software (Image Reader LAS-1000 Lite, Fuji Photo Film Co. Ltd.).

### 2.14. Microarray Expression Analysis of hUCB-MSCs

Five micrograms of total RNA from hUCB-MSCs was hybridized to the Human-GE 4x44K v2 Microarray (human whole genes; Agilent Technologies, Santa Clara, CA). Two types of hUCB-MSCs were analyzed and compared: N3 (cells at passage 3, normal pregnancy) and P3 (cells at passage 3, preeclampsia). The standard protocol used for sample preparation and microarray processing is available from Agilent Technologies. Expression data were analyzed using Agilent's GeneSpring GX software (Genomictree Inc., Daejeon, Korea).

### 2.15. Pathway Network Analysis of Differentially Expressed Genes (DEGs)

The functional interactions between differentially expressed genes (DEGs) were analyzed by GeneMANIA web server [53]. The GO term was used to create the interaction network between the DEGs and additional genes by using human as a source species. DEGs were mapped to the GeneMANIA to investigate how these genes interact with each other and additional genes that are related to a set of query genes by using a very large set of functional interaction data. By integrating these relationships, a network between DEGs and additional related genes was constructed for intersection of DEG sets N3 vs. P3. To confirm the gene network created with DEGs, the GO term enrichment analysis was performed among the genes in the network.

### 2.16. Statistical Analysis

Reported data are the mean ± standard deviation (SD). Patients' characteristics, cell population doubling time, densitometric values for SA-*β*-gal-positive cells, telomerase activity, and ROS activity were compared between groups by a Mann-Whitney *U* test. Other variables, including the cell number, were compared by Student's *t*-test using SPSS, version 12.0 (SPSS, Chicago, IL). A *p* value less than 0.05 was considered statistically significant.

## 3. Results

### 3.1. Clinical Characteristics of Participants in the Normal and Preeclampsia Groups

The clinical characteristics of the patients who provided cord blood for the study are presented in Table 2. There was no significant difference in maternal age or gestational age at delivery between the normal and preeclampsia groups. Birth weight in the preeclampsia group was significantly lower than that in the normal group. Systolic and diastolic blood pressure was significantly higher in the preeclampsia group than in the normal group.

### 3.2. Differentiation and Characterization of hUCB-MSCs

**Figure 1:**
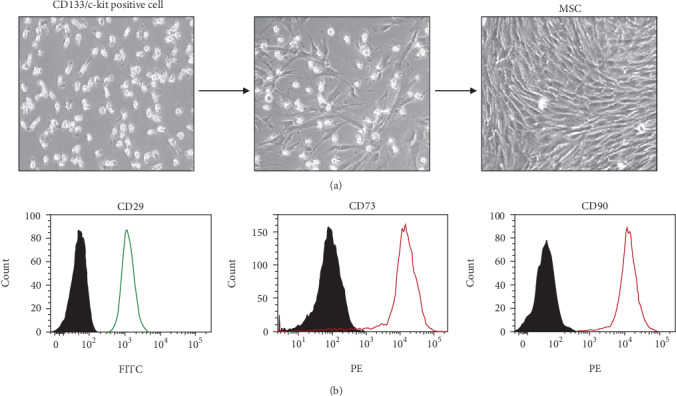
Differentiation of CD133/c-kit-positive cells from human umbilical cord blood of the normal group (*n* = 20) into mesenchymal stem cells (MSCs). (a) Differentiation of CD133/c-kit cells into MSCs. (b) MSCs derived from human umbilical cord blood were positive for the MSC markers CD29, CD73, and CD90. FITC: fluorescein isothiocyanate; PE: phycoerythrin.

**Figure 2:**
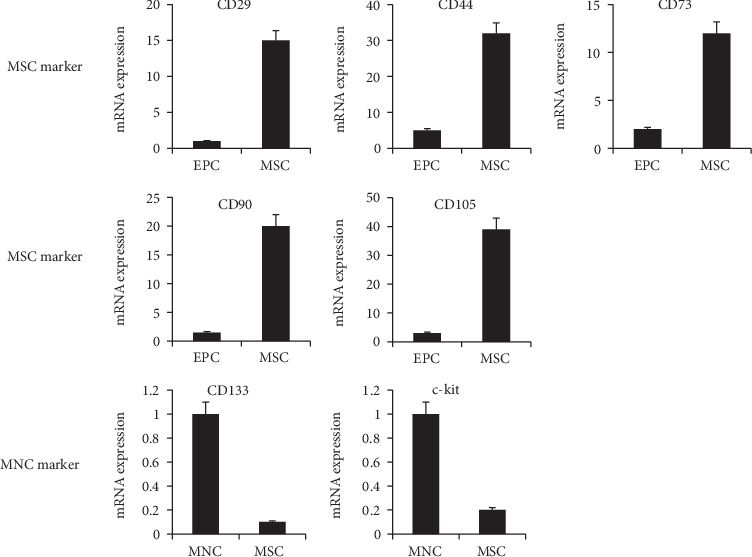
Characterization of CD marker profile of MCSs derived from human umbilical cord blood (hUCB-MSCs) of the normal group (*n* = 20) by RT-qPCR. hUCB-MSCs were positive for the expression of CD29, CD44, CD73, CD90, and CD105 but negative for CD133 and c-kit.

**Figure 3:**
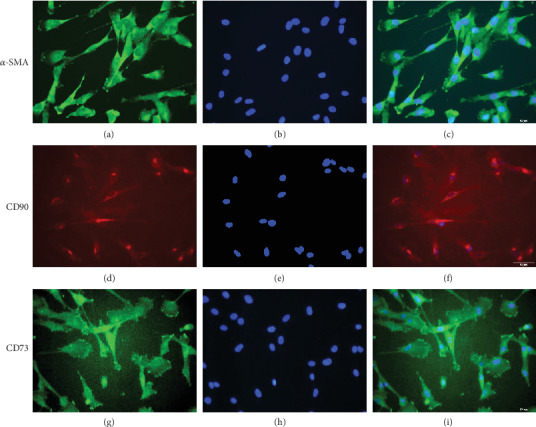
Marker protein expression in hUCB-MSCs. hUCB-MSCs from the normal group (*n* = 20) were stained for *α*-SMA (a, c), CD90 (d, f), and CD73 (g, i). Protein expression is indicated by green and red fluorescence, and nuclei are indicated by DAPI staining (blue; b, e, h).

Among the MNCs obtained from umbilical cord blood, CD133/c-kit-positive cells were selected and differentiated into MSCs. MSCs differentiated from CD133/c-kit-positive cells were characterized with morphology (Figure 1(a)), flow cytometric analysis (Figure 1(b)), semiquantitative RT-qPCR (Figure 2), and immunofluorescence staining (Figure 3). hUCB-MSCs obtained from 20 normal pregnancies were used for MSC characterization analysis.

### 3.3. In Vitro Differentiation Studies of hUCB-MSCs

**Figure 4:**
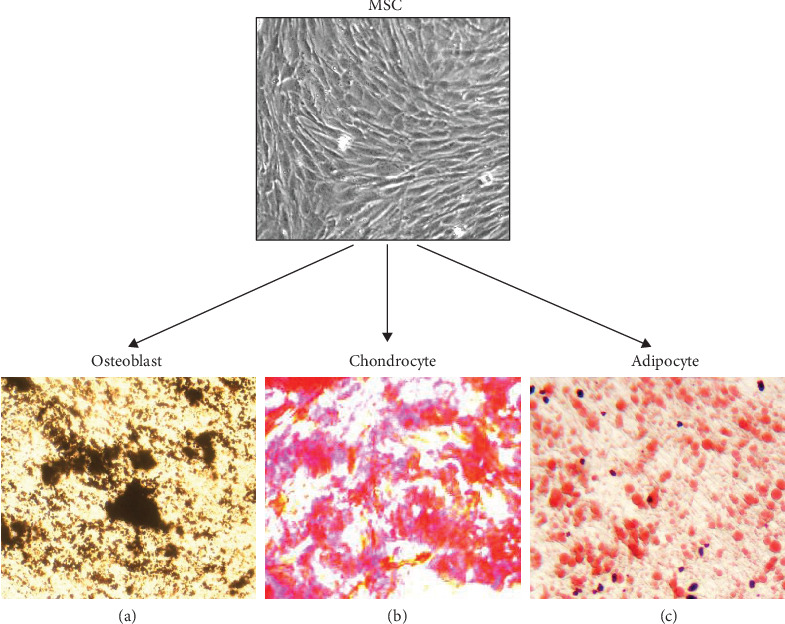
Multilineage differentiation capacity of hUCB-MSCs. The MSCs obtained from umbilical cord blood of the normal group (*n* = 20) were investigated for their in vitro multilineage differentiation capacity ((a) osteogenesis; (b) chondrogenesis; (c) adipogenesis).

Osteogenic differentiation of hUCB-MSCs was confirmed by the detection of an osteogenic phenotype consisting of increased expression of alkaline phosphatase and by the deposition of a silver-stained mineralized matrix (Figure 4(a)). Chondrogenic differentiation was confirmed by the formation of a sphere in the micromass culture and the secretion of cartilage-specific proteoglycans stainable with Safranin O (Figure 4(b)). Adipogenic differentiation of the cells was demonstrated by the accumulation of neutral lipid vacuoles stained by Oil Red O (Figure 4(c)).

### 3.4. Decreased Proliferative Potential of hUCB-MSCs from Preeclampsia

**Figure 5:**
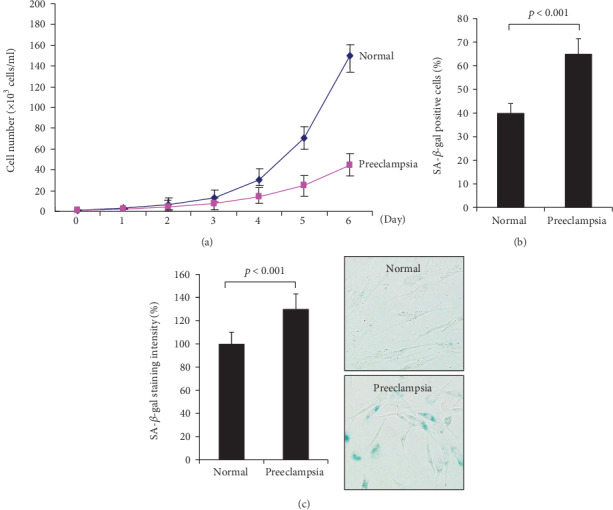
Increased senescence of hUCB-MSCs from women with preeclampsia. (a) Proliferation assay of hUCB-MSCs from the two groups. After 6 days, the number of cells was significantly lower in cultures of hUCB-MSCs derived from women with preeclampsia than in cultures of hUCB-MSCs derived from women with normal pregnancy. Data are the mean ± SD. (b) The number of SA-*β*-gal-positive cells was counted from at least 200 cells. The percentage of cells that were clearly SA-*β*-gal-positive was significantly higher in the preeclampsia group (*n* = 10) than in the normal group (*n* = 10). (c) The staining intensity of SA-*β*-gal-positive cells was determined by densitometry. The relative staining intensity was significantly higher in the preeclampsia group. Values are the mean ± SD. *p* < 0.001 by Mann-Whitney *U* test.

To compare the proliferative ability of hUCB-MSCs from women with normal pregnancy and preeclampsia, a proliferation assay was performed. It was apparent that the proliferation of hUCB-MSCs from women with preeclampsia was significantly reduced in comparison with that of normal pregnancy (Figure 5(a)). These data demonstrate that hUCB-MSCs from women with preeclampsia have a much lower expansion potential than those from women with normal pregnancy.

### 3.5. Increased Senescence of hUCB-MSCs from Women with Preeclampsia

A SA-*β*-gal assay of hUCB-MSCs obtained from women with normal pregnancy or preeclampsia was performed to assess the characteristics of cellular aging in vitro. The number of SA-*β*-gal-positive cells was significantly higher in hUCB-MSCs from women with preeclampsia (64.5%; range, 58.8-70.2%) than in those from women with normal pregnancy (39.8%; range, 35.0-44.6%; *p* < 0.001) (Figure 5(b)). The mean staining intensity was significantly higher in the preeclampsia group than in the normal group (129.5 ± 12.3% vs. 100.0 ± 11.1%; *p* < 0.001) (Figure 5(c)).

### 3.6. Decreased Telomerase Activity in hUCB-MSCs from Women with Preeclampsia

**Figure 6:**
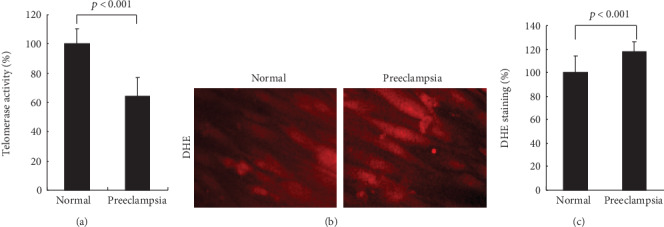
Decreased telomerase activity of hUCB-MSCs from women with preeclampsia. (a) Quantitative analysis of preeclampsia-related alterations of telomerase activity. Mean telomerase activity of hUCB-MSCs was significantly lower in the preeclampsia group (*n* = 10) than in the normal group (*n* = 10). Values are the mean ± SD. *p* < 0.001 by Mann-Whitney *U* test. (b) Representative photomicrographs showing hUCB-MSCs stained for ROS with DHE, from women with normal pregnancy and preeclampsia. (c) Quantification of DHE intensity of hUCB-MSCs. The DHE staining was determined by densitometry (*n* = 10 in each group). Relative DHE intensity was significantly higher in the preeclampsia group. Values are the mean ± SD. *p* < 0.001 by Mann-Whitney *U* test.

Preeclampsia-related alterations of telomerase activity in hUCB-MSCs were evaluated. Telomerase activity was lower by 40% in hUCB-MSCs from the preeclampsia group compared with those from the normal group (Figure 6(a)). In addition, ROS can bring about cellular senescence, apoptosis, or carcinogenesis, and ROS-induced cellular damage also contributes to stem cell aging [54]. As shown in Figures 6(b) and 6(c), fluorescence of ROS by DHE was increased in hUCB-MSCs from women with preeclampsia.

### 3.7. Comparisons of Gene Expression in hUCB-MSCs from the Normal and Preeclampsia Groups Using Microarray Analysis

hUCB-MSCs obtained from 2 normal pregnancies and 2 preeclampsia were used for comparison of gene expression including the gene expression pattern using microarray, hierarchical cluster analysis of differentially expressed genes, Gene Ontology classification, and pathway network analysis between two groups.

**Figure 7:**
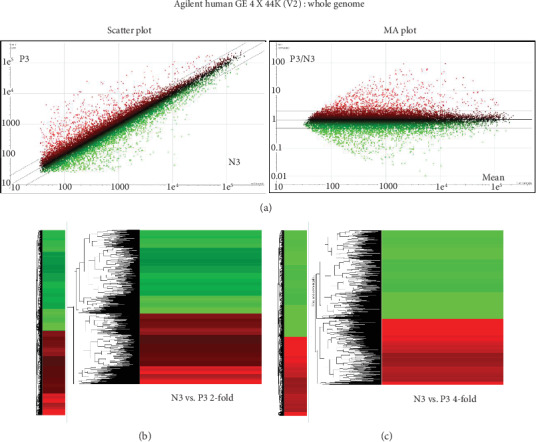
Microarray analysis of hUCB-MSCs from the normal and preeclampsia groups. (a) The scatter plot and MA plot show the DEGs between N3 and P3 cells. In the scatter plot, the median line indicates no difference in signal intensity between the two groups. The upper gray line indicates a twofold higher signal intensity, and the lower gray line indicates a twofold lower signal intensity for hUCB-MSCs from the preeclampsia group as compared to the normal group. In the MA plot, the median line represents N signal/P signal = 1. The upper gray line represents N signal/P signal = 2, and the lower gray line represents N signal/P signal = 0.5. N3: hUCB-MSCs at passage 3, normal pregnancy; P3: hUCB-MSCs at passage 3, preeclampsia. (b, c) Hierarchical cluster analysis of differentially expressed genes (DEGs) between N3 and P3 cells. Red represents upregulated gene clusters, and green represents downregulated gene clusters. The panels show genes up- or downregulated twofold (b) or fourfold (c) in P3 (versus N3) cells. N3: hUCB-MSCs at passage 3, normal pregnancy; P3: early hUCB-MSCs at passage 3, preeclampsia.

After data processing, expression profiles were analyzed by scatter plot and MA (log ratio and mean) plot (Figure 7(a)). hUCB-MSCs at passage 3 from the normal group (N3) were compared with cells at passage 3 from the preeclampsia group (P3). In the plots, red spots represent genes with higher signal intensity in hUCB-MSCs from the preeclampsia group than in the hUCB-MSCs from the normal group. Green spots represent decreased signal intensity. The scatter plot and MA plot show the differentially expressed genes (DEGs) between N3 and P3.

Expression of genes was compared between hUCB-MSCs from the normal and preeclampsia groups, and genes were clustered by the expression pattern (Figures 7(b) and 7(c)). In the comparison between N3 and P3 cells, twofold and fourfold differences in expression (either up- or downregulation) were detected for 2684 genes and 659 genes, respectively.

**Figure 8:**
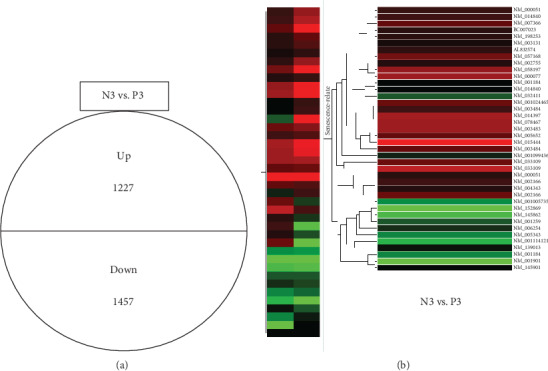
Comparison and cluster analysis of differentially expressed genes (DEGs) between N3 and P3. (a) Twofold upregulated genes and twofold downregulated genes were identified between N3 and P3. N3: hUCB-MSCs at passage 3, normal pregnancy; P3: hUCB-MSCs at passage 3, preeclampsia. (b) Dendrogram showing differential senescence-related gene expression in hUCB-MSCs from women with normal pregnancy or preeclampsia. Differentially expressed genes (DEGs) related to senescence were compared between N3 and P3 cells. N3: early hUCB-MSCs at passage 3, normal pregnancy; P3: early hUCB-MSCs at passage 3, preeclampsia.

Genes differentially expressed between N3 and P3 cells were compared to identify specific DEGs in each group. Twofold up- and downregulated genes were used in these comparisons. We identified 1227 upregulated and 1457 downregulated genes that were common between N3 and P3 (Figure 8(a)). After the hierarchical clustering of senescence-related DEGs, we constructed a dendrogram to display the clusters (Figure 8(b)). Forty senescence-related DEGs were identified.

### 3.8. Functional Categorization and Pathway Network Analysis of Senescence-Related Differentially Expressed Genes (DEGs)

The Gene Ontology (GO) term was used to create the interaction network between the senescence-related DEGs and additional genes by using human as a source species. The relationship between the genes in the network includes coexpression, physical interactions, pathways, colocalization, and protein domain similarity. The list of senescence-related DEGs was enriched for certain GO terms. Among the GO terms that have a significant relationship with senescence, determined by low false discovery rate (FDR), were genes associated with the cell cycle, which showed a very strong relationship with the selected genes (Table 3).

**Figure 9:**
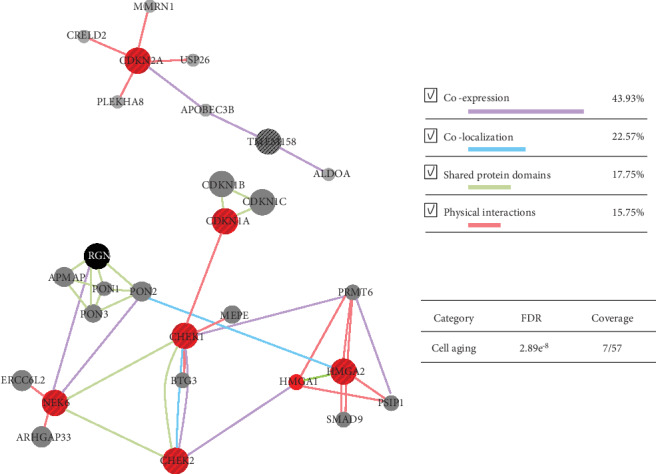
Network analysis of senescence-related differentially expressed genes (DEGs). Genes represented by a large circle are senescence-related differentially expressed genes (DEGs) obtained from microarray analysis. Red ones are genes related with senescence as well as cell aging. FDR: false discovery rate.

Among 40 senescence-related DEGs, we identified eight genes with filtering conditions of differential expression with more than twofold in N3 vs. P3 and then performed a GO term enrichment analysis with these genes. In particular, we investigated any relationship of those genes. The eight senescence-related DEGs had two networks. GeneMANIA network analysis for those genes suggested enrichment of 7 genes related to the “cell aging” GO term, including NM_078467, NM_058197, NM_001114121, NM_145862, NM_003483, NM_014397, and NM_003483 (GenBank with a large red circle in Figure 9). Most of relations between genes were coexpressed. In the network, GO term “cell aging” is significantly enriched with FDR-corrected *p* value 2.89*e*^−8^. Among 57 genes related to “cell aging,” seven genes were covered.

## 4. Discussion

Preeclampsia is a disease characterized by pregnancy-induced hypertension and proteinuria that affects 2-8% of all pregnancies [31, 32, 34]. In women with this condition, the intrauterine environment is modified by changes in signaling patterns and substrate transport to the fetus [55, 56]. This modification can lead to fetal growth restriction and increased susceptibility to diseases later in life, such as cardiovascular, endocrine, nutritional, metabolic, and blood-related disease. The change of the intrauterine environment made in preeclampsia can also be induced by the circulating factors in umbilical cord blood. The fetal circulation during preeclampsia may be associated with an increase in circulating antiangiogenic factors such as sFlt-1 (soluble fms-like tyrosine kinase 1) and soluble endoglin or reduced expression and activity of proangiogenic signals such as vascular endothelial growth factor or adenosine [57, 58]. Some studies found higher concentration of protein oxidation product (ex, protein carbonyl) and an increase of oxidative stress and lipid peroxidation in the cord blood of preeclamptic pregnancy compared to normotensive pregnancy [59]. The umbilical serum level of inflammatory markers (interleukin-6, interleukin-8, and tumor necrosis factor-alpha) in pregnancies complicated by preeclampsia was significantly increased compared with normal pregnancies [60]. Because of these pathologic conditions in the umbilical cord blood of women with preeclampsia, we hypothesized that circulating hUCB-MSCs in preeclamptic pregnancy cannot be functionally impaired.

Recent studies showed that in women with preeclampsia, cord blood endothelial progenitor cells (EPCs) or circulating endothelial colony-forming cells were decreased and functionally perturbed, and this might contribute to an increased risk of future cardiovascular events [20, 61]. However, fetal growth restriction and diseases occurring after birth cannot be explained simply by alteration of endothelial stem cells derived from umbilical cord blood. There are many kinds of multipotent stem cells in umbilical cord blood including EPCs and MSCs. Because MSCs can self-renew, have a high proliferative capacity, and can differentiate into various cell types such as chondrocytes, osteocytes, adipocytes, myocytes, and neurons, hUCB-MSCs are highly likely to be functionally impaired in women with preeclampsia. In the present study, it was shown that hUCB-MSCs obtained from women with preeclampsia were less proliferative and more senescent than cells from women with normal pregnancy, and many senescence-related DEGs were identified by analysis of gene expression profiles.

Despite being a promising tool in regenerative medicine, MSCs remain controversial. This is because the clinical usefulness of MSCs, resulting from their multipotency and wide accessibility, is countered by their finite proliferative ability. Many factors affect the proliferation and senescence of MSCs in vitro, such as replicative senescence, donor age, and culture condition [62–64]. Therefore, many studies of MSCs have generated conflicting data showing a tremendous variance in growth potential. The results of this study showed that hUCB-MSCs from women with preeclampsia were poorly proliferative, more senescent, and had decreased telomerase activity and increased ROS activity. These preeclampsia-associated changes in hUCB-MSCs were not related to donor age, replicative senescence, and culture condition. Therefore, there may be an unknown pathway associated with MSC senescence. Hierarchical clustering by compared gene expression analysis identified 40 senescence-related DEGs. These genes will be potential research targets in the future studies of MSCs.

At present, MSCs are extensively characterized in a culture-expanded state, and relatively little is known of their biological properties in vivo. Generally, ROS can bring about cellular senescence, apoptosis, or carcinogenesis. ROS-induced cellular damage also contributes to stem cell aging [54]. A recent study showed that human MSCs had high resistance to oxidative stress-induced death, which correlated with a low level of intracellular reactive species due to effective ROS scavenging, constitutive expression of enzymes required to manage oxidative stress, and high levels of total intracellular glutathione [65]. Also, many studies suggest that telomeres and telomerase have important roles in senescence in vitro and in vivo [66]. Telomerase, a ribonucleoprotein complex containing a template RNA subunit, extends telomere length by adding telomeric repeats to the chromosome ends [67]. The high production of ROS results in a state of oxidative stress, which subsequently leads to senescence with the shortening of telomeres [68]. So, telomerase has telomere-independent antiapoptotic, cytoprotective, and proproliferative effects of telomerase or protection of mitochondrial DNA against oxidative stress in addition to telomere elongation [69]. In this study, all cells were cultured under the same normoxic conditions. Nevertheless, hUCB-MSCs from women with preeclampsia were consistently more senescent and had higher ROS activity and lower telomerase activity than in women from the normal group. These findings can give explanations for the senescence of hUCB-MSCs from preeclampsia.

Comparison and cluster analysis of genes differentially expressed between N3 and P3 cells showed that 1227 upregulated and 1457 downregulated DEGs were common to both sets and were related to the reduced function of hUCB-MSCs from women with preeclampsia. Through intersection analysis of microarray data, we eliminated the false up- or downregulated DEGs, which could have caused misinterpretation of the microarray data. Through GO term categorization and pathway network analysis, we confirmed that the selected genes are highly related to proliferation and cell cycle, all of which are the important causes or effects of cellular senescence. The senescence-related DEGs in two networks may be mainly associated with increased senescence of preeclamptic hUCB-MSCs. Those genes showed a network with a coexpression pattern, and some of them are definitely involved in the cell aging process. The senescence-related genes identified in this study can be further analyzed in many different ways.

A potential new pathway for MSC senescence suggested in our results should be studied through the verification and analysis of senescence-associated genes. If hUCB-MSC markers related to intrauterine growth restriction and diseases occurring later in life are found, these pathological consequences of preeclamptic pregnancy may be resolved. Future studies of MSCs should focus on the effective promotion of long-term cell expansion, identification of pathways relevant to replicative exhaustion, and maximum growth capability without loss of the ability to differentiate.

## 5. Conclusion

In conclusion, the pathologic condition in the umbilical cord blood of women with preeclampsia causes fetal growth restriction and increased susceptibility to diseases later in life. hUCB-MSCs obtained from women with preeclampsia are poorly proliferative and more senescent, have increased ROS activity and decreased telomerase activity compared with cells from women with normal pregnancy, and are related with many senescence-related DEGs identified by analysis of gene expression profiles. Because these preeclampsia-associated changes in hUCB-MSCs are not related to donor age, replicative senescence, and culture condition, another pathway associated with MSC senescence should be studied in the near future. 40 senescence-related DEGs identified in this study will be potential research targets in the future studies of MSCs.

## Figures and Tables

**Table 1:** Primer sequences specific to the target genes.

Gene	Direction	Sequence
CD133	Sense	5′-CCCGCAGGAGTGAATCTTTT-3′
	Antisense	5′-AGGAAGGACTCGTTGCTGGT-3′
c-kit	Sense	5′-TCTCTTTAGGAAGCAGCCCC-3′
	Antisense	5′-ACATTTCAGCAGGTGCGTGT-3′
CD29	Sense	5′-GTAGCTGGTGTGGTTGCTGG-3′
	Antisense	5′-TGTCCCATTTGGCATTCATT-3′
CD44	Sense	5′-GGTGCATTTGGTGAACAAGG-3′
	Antisense	5′-CACCCCAATCTTCATGTCCA-3′
CD73	Sense	5′-TGGATGGCTCCTCTCAATCA-3′
	Antisense	5′-GCACATGGATACGTGGTTCC-3′
CD90	Sense	5′-TCTCCTCCCAGAACGTCACA-3′
	Antisense	5′-GAGAGGGAGAGCAGGAGCAG-3′
CD105	Sense	5′-GAGGCGGTGGTCAATATCCT-3′
	Antisense	5′-GTAGAGGCCCAGCTGGAAAG-3′
GAPDH	Sense	5′-ATGGGGAAGGTGAAGGTCG-3′
	Antisense	5′-GGGGTCATTGATGGCAACAATA-3′

**Table 2:** Clinical characteristics of study participants.

Variable	Normal group (*n* = 30)	Preeclampsia group (*n* = 28)
Maternal age (years)	31 ± 2.1	32 ± 2.5
Gestational age at delivery (weeks)	36 ± 1.6	35 ± 1.7
Birth weight (kg)	3.14 ± 0.31	2.81 ± 0.48^∗∗^
Systolic blood pressure (mmHg)	115 ± 5	173 ± 12^∗∗^
Diastolic blood pressure (mmHg)	69 ± 6	106 ± 9^∗∗^
Proteinuria	None	28/28^∗^
Nulliparity (*n*, %)	23 (76.7)	21 (75.0)^†^
Indications of cesarean section (*n*, %)		
Previous cesarean section	7 (23.3)	7 (25.0)
Previous myomectomy	12 (40.0)	3 (10.7)
Breech presentation	9 (30.0)	13 (46.4)
Transverse lie	2 (6.7)	5 (17.9)
Methods of anesthesia (*n*, %)		
General anesthesia	19 (63.3)	20 (71.4)
Regional anesthesia	11 (36.7)	8 (28.6)

Data are the mean ± SD. ^∗^≥2+ on a urine dipstick test. ^∗∗^Mann-Whitney *U* test, *p* < 0.05 (statistically significant). ^†^Pearson chi-squared (*χ*^2^) test, *p* > 0.05.

**Table 3:** Gene Ontology classification of the senescence-related differentially expressed genes in hUCB-MSCs from the normal and preeclampsia groups.

GO name	List numbers	Total numbers	FDR
Cell cycle phase	122	414	1.78*e* − 37
Cell cycle	170	776	2.96*e* − 34
M phase	103	329	4.64*e* − 34
Cell cycle process	140	565	5.40*e* − 34
Mitotic cell cycle	107	370	4.92*e* − 32
M phase of mitotic cell cycle	80	224	7.77*e* − 31
Nuclear division	79	220	1.23*e* − 30
Mitosis	79	220	1.23*e* − 30
Organelle fission	79	229	2.74*e* − 29
Cell division	79	295	2.02*e* − 21
DNA replication	56	190	2.30*e* − 17
Chromosome segregation	35	81	1.11*e* − 16
Regulation of cell cycle	73	331	8.05*e* − 15
DNA metabolic process	95	506	2.13*e* − 14
DNA packaging	37	117	1.25*e* − 12
Protein-DNA complex assembly	30	91	7.44*e* − 11
Cell cycle checkpoint	30	91	7.44*e* − 11
Regulation of mitotic cell cycle	39	152	2.96*e* − 10
Response to DNA damage stimulus	68	373	6.05*e* − 10
Spindle organization	20	45	6.12*e* − 10
Chromatin assembly	28	87	6.55*e* − 10
Chromosome organization	81	485	8.97*e* − 10

List numbers: numbers of DEGs belonging to specific GO terms; total numbers: total numbers of genes belonging to specific GO terms; FDR: false discovery rate.

## Data Availability

The data used to support the findings of this study are available from the corresponding author upon request.
